# Serological Evidence of Exposure to Peste des Petits Ruminants in Small Ruminants in Rwanda

**DOI:** 10.3389/fvets.2021.651978

**Published:** 2021-03-04

**Authors:** Anselme Shyaka, Marie Aurore Ugirabe, Jonas Johansson Wensman

**Affiliations:** ^1^School of Veterinary Medicine, College of Agriculture, Animal Sciences and Veterinary Medicine, University of Rwanda, Nyagatare, Rwanda; ^2^Department of Clinical Sciences, Swedish University of Agricultural Sciences, Uppsala, Sweden

**Keywords:** small ruminants, Rwanda, PPR, seroprevalence, transboundary diseases

## Abstract

The status of Peste des Petits Ruminants (PPR) in Rwanda is unknown, despite its prevalence in neighboring countries. A cross-sectional sampling of goats and sheep was carried out in five districts of Rwanda located closer to neighboring countries endemic to PPR. Serum samples were analyzed using a commercial ELISA, to detect antibodies to PPR virus (PPRV). Sixty-eight samples [14.8, 95% Confidence Interval (CI): 11.7–18.4] were seropositive for PPR, of which 17.4% (95% CI: 11.6–24.6; 25/144) were from sheep, whereas 13.6% (95% CI: 10.0–17.9; 43/316) were from goats. Seropositivity ranged from 8.9 to 17.3% (goats) and from 10.5 to 25.8% (sheep) in sampled districts. Seropositivity was slightly higher in males than females in both goats (15.7 vs. 12.4%) and sheep (17.7 vs. 17.1%), and were significantly marked in goats and sheep aged more than 15 months (goats: 17.9, 95% CI: 12.9–24.0; sheep: 22.2, 95% CI: 14.1–32.2) than those between 6 and 15 months (goats: 6.1, 95% CI: 2.5–12.1; sheep: 9.3, 95% CI: 3.1–20.3). Sampling was non-randomized and results are not representative of the true prevalence of PPR antibody in small ruminants. Thus, data does not allow to fully discuss the findings beyond the presence/absence certitude and the comparisons made must be interpreted with caution. The presence of specific antibodies to PPRV may, however, be linked to one or a combination of following scenarios: (1) prevalence and persistence of PPRV in sampled regions which would cause low level of clinical cases and/or mortalities that go unnoticed; (2) introduction of PPRV to herds through movements of livestock from neighboring infected countries, and/or (3) events of disease outbreaks that are underreported by farmers and veterinarians. In addition to strengthen veterinary surveillance mechanisms, further studies using robust sampling methods and integrating livestock and wildlife, should be carried out to fully elucidate PPR epidemiology in Rwanda.

## Introduction

Livestock diseases are recognized global threats to food supply and to livestock industry specifically (1). Peste des Petits Ruminants Virus (PPRV) is a member of the family *Paramyxoviridae*, genus *Morbillivirus*, species *Small ruminant morbillivirus* (2). It primarily affects goats and sheep, but also other domestic animals such as cattle, pigs and camels as well as various wildlife ungulates (3, 4), through contact with infected animals, or indirectly through fecal and/or mucosal secretions (5). The disease caused, Peste des Petits Ruminants (PPR), is highly contagious and is characterized by acute clinical signs in goats and sheep, as well as in wild ruminants (6–8). PPR is associated with a case fatality rate of 15.5% (8) that can reach up to 80–100% in naïve herds (9). PPR is recognized as the most widely distributed infectious disease of domestic small ruminants and wildlife ungulates, and is endemic in most countries of Africa, Middle East and Asia (10). It can negatively impact countries' economy and increase poverty in rural settings where small ruminants are mostly concentrated. In fact, PPR-associated losses are estimated at USD 1.2–1.7 billion annually and a third of this financial burden occurs in Africa (10). In addition, PPR constitutes a growing challenge to biodiversity and wildlife conservation (8, 11).

PPR has affected most countries in East Africa since the last 5 decades and confirmation of the first outbreak in Sudan in 1971–1972 (12), followed by further outbreak reports from Ethiopia in 1989–1990 (13). In Uganda, the major PPR outbreak was reported in 2006–2008, in Karamoja region along with a similar report in neighboring Kenya (14, 15). However, previous reports had suggested presence of PPR through seroprevalence studies carried out in the 1980s in Uganda and Kenya (16), in the 2000s in Uganda (17) and an outbreak reported in Uganda in 2003 (18). In addition, antibodies to PPRV were also detected in Ugandan wildlife in 2004 (19), probably as a consequence of spillover of the virus from livestock. Tanzania had its first confirmation of PPR in 2008 (20), with retrospective serological evidence of earlier circulation (17) and PPR is currently considered endemic, including in Kagera and Kigoma regions close to Rwanda (21). In neighboring Burundi, a first outbreak of PPR occurred in December 2017 to February 2018 (22), but retrospective serological analysis detected antibodies to the virus in samples collected in early 2017. A more recent study highlighted circulation of PPRV in livestock and wildlife living in eastern DRC and western Uganda (19). This brief history shows that PPR has had endemic events in various regions of east Africa surrounding Rwanda, with periodic outbreaks and circulation of the virus in various susceptible animals including detection in wildlife. Phylogenetic analyses of circulating viruses, showed that PPRV lineage II, III, and IV are prevalent in DRC, Uganda, Tanzania, Kenya, and Burundi (22–26).

Rwanda status vis-à-vis PPR is unknown (27). In fact, there has never been any empirical study to establish the prevalence of PPR in the country, despite its occurrence in the neighboring countries (9, 19, 22, 24, 28–31). The presence of this disease in the regional countries, transboundary movements of livestock passing through official and non-official entry/exit points and important wildlife species constitute potential factors for PPRV introduction in the country. In order to strengthen prevention mechanisms against PPR in Rwanda toward eradication of PPR by 2030, it is important to establish systems of surveillance as part of the stage 1 or “Assessment stage” of the Global Strategy for the Control and Eradication of PPR (10). An efficient surveillance stage would give insights on whether the disease is present and passes unnoticed, and provide information for the next steps toward the elimination of PPR. Such surveillance mechanisms must adopt strategies for the control of PPR in susceptible domestic and wild animals in Rwanda, in order to establish presence, circulation and persistence of the virus. This study investigated the prevalence of specific antibodies to PPRV and aimed at providing baseline data that can be used by concerned regulatory bodies and stakeholders to scale up PPR investigation in Rwanda and set up adequate prevention measures.

## Materials and Methods

### Ethical Approval

Ethical approval for this study was obtained from the College of Agriculture, Animal Sciences and Veterinary Medicine, University of Rwanda (Ethical approval reference: 025/17/DRIPGS). Approved consent forms were distributed and signed prior to the interviews and sampling of animals. Sampling of animals was done following the protocols in conformity with the World Organization for Animal Health (OIE) Terrestrial Animal Health Code 2012 (use of animals in research and education).

### Area Description and Study Design

**Figure 1:**
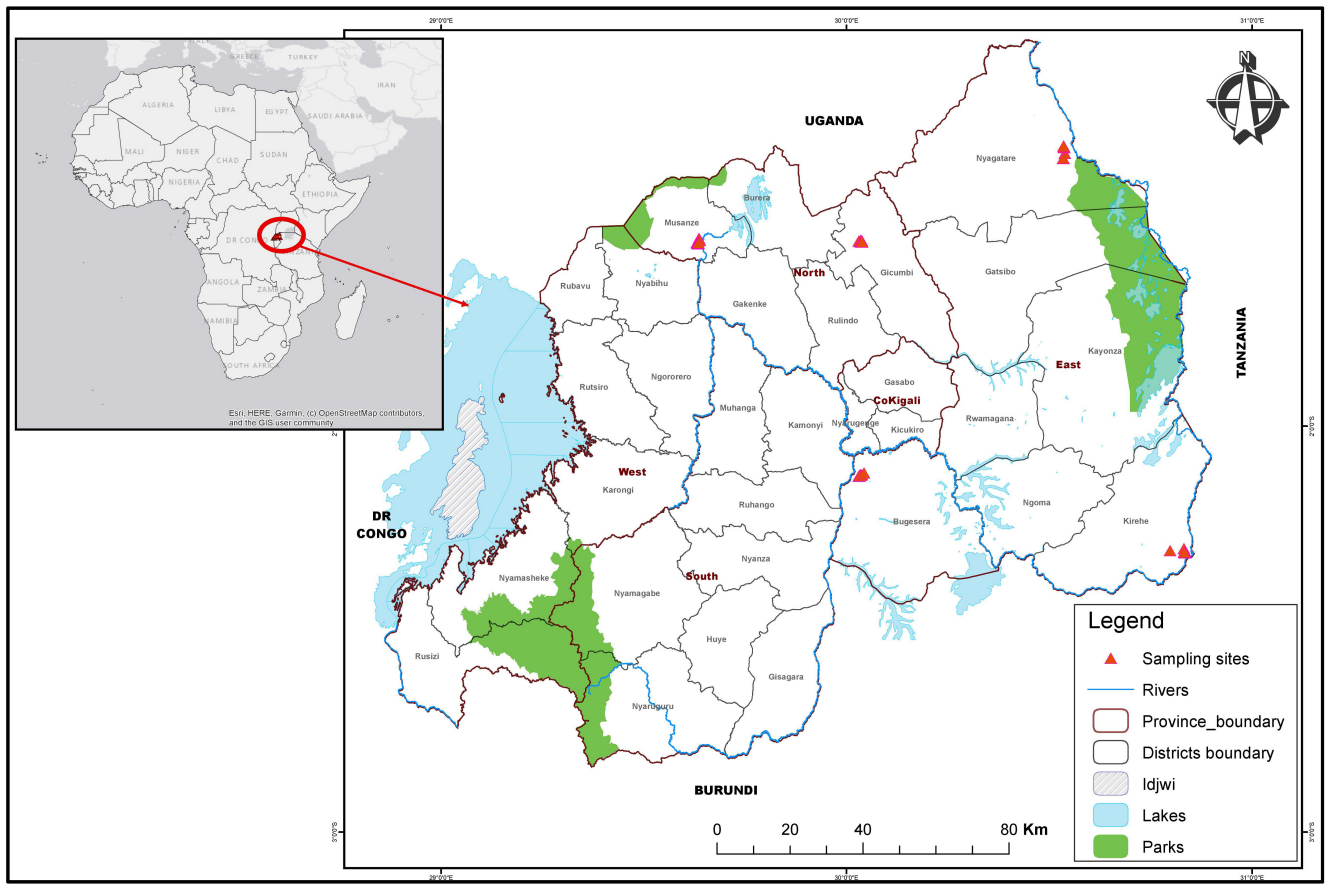
Map of Rwanda districts highlighting sampling sites in East and North areas. The map shows administrative boundaries of districts and provinces. The red triangles highlight sampling sites in Nyagatare, Kirehe and Bugesera districts of the Eastern Province as well as Musanze and Gicumbi districts in the Northern Province.

Samples were collected from Bugesera, Kirehe, and Nyagatare districts of the eastern Province in Rwanda, and from Gicumbi and Musanze districts of the Northern Province (Figure 1). The five sites sampled are relatively close to borders with Uganda to the north, Tanzania to the east and Burundi to the south.

This cross-sectional study was conducted during a period of 3 months, from January to March 2019. Non-probability convenience samples were collected in farms located in the study area, under guidance of local veterinarians. In addition, a small questionnaire was used to collect information related to animal sampled, herd management and general animal health at the farm and its surrounding. Goats and sheep, apparently healthy, non-vaccinated against PPR and having more than 6 months of age, were recruited into the study. To accurately estimate the age of goats and sheep, the age dentition method was used, according to methods described elsewhere (32, 33). To calculate the sample size, we used a recommended formula for estimating the adequate sample size in prevalence study (34). Thus, assuming a large and homogenous population size with an estimated 50% prevalence (P) that optimizes the sample size, with a confidence level set at 95% and ±5% precision, the formula $n=\frac{z^{2}P\left(1-P\right)}{d^{2}}$ recommended 385 samples. Finally, 460 blood samples were drawn from jugular veins of apparently healthy goats and sheep, using Vacutainer needles and sterile plain tubes. Samples were allowed to clot overnight in order to maximize sera collection, which were harvested following a centrifugation at 3,000 rpm for 5 min. The sera were then stored at −20°C until screening was done.

### Screening of the Samples

The screening for the presence of PPR was done to detect antibodies to the nucleoprotein of Peste the Petits Ruminants virus, using a commercial competitive ELISA (cELISA) kit (ID screen^®^ PPR competition, IDvet Genetics, Grabels, France) according to the manufacturer's instructions. The sensitivity and specificity of this cELISA in sheep and goats, is estimated at 94.5 and 99.4%, respectively, compared to virus neutralization assay (35). The optical densities (ODs) were read using a Thermo Scientific™ Multiskan™ FC Microplate Photometer at a wavelength of 450 nm and the results were expressed as sample positivity percentage (S/N %). Samples were considered positive if the S/N % were ≤50%, negative if ≥60% or doubtful if it was between 50 and 60%. Since the sampled small ruminants had no clinical signs of PPR, doubtful results were finally considered as negative.

### Statistical Analyses

The true prevalence in positive animals and herds was estimated by adjusting the apparent prevalence obtained from cELISA results to the sensitivity and specificity of the test, as described by Rogan and Gladen (36). In addition, in order to test for independence between two variables, univariable analysis was done using chi-square test. All the statistical analyses were carried out using R Statistical Software (version 3.6.3; R Foundation for Statistical Computing, Vienna, Austria).

## Results

### Characteristics of Study Respondents

The descriptions below for study participants and characteristics of farms and small ruminants sampled can be found in Table 1.

**Table 1:** Characteristics of study respondents and farms sampled (*n* = 45).

**Variables**		**Frequency (%)**
District	Nyagatare	6 (10.5)
	Kirehe	18 (31.6)
	Bugesera	7 (12.3)
	Gicumbi	12 (21.1)
	Musanze	14 (24.6)
Gender	Female	19 (33.3)
	Male	38 (67.7)
Age quintiles (years)	<20	5 (8.8)
	21–30	3 (5.3)
	31–40	11 (19.3)
	42–50	17 (29.8)
	>50	21 (36, 8)
Education	No formal education	17 (29.8)
	Primary	28 (49.1)
	Secondary	12 (21.1)
Experience in animal husbandry (years)	<1	1 (1.8)
	1–5	24 (42.1)
	6–10	8 (14.0)
	>10	24 (42.1)
Types of small ruminant owned	Goats only	36 (63.2)
	Sheep only	9 (15.8)
	Goats and Sheep	12 (21.1)
Farming system	Zero-grazing	35 (61.4)
	Semi-zero-grazing	9 (15.8)
	Open grazing	13 (22.8)
Small ruminants disease history	Occurrence of abortions at sampled farms	19 (33.3)
	Occurrence of death at sampled farms	24 (42.1)
Small ruminants disease history in neighboring farms	Report of abortions in neighboring farms	12 (21.1)
	Report of death in neighboring farms	21 (36.8)

In total, this study reached 57 households distributed in the 5 districts targeted by this research. Participants were composed of 19 females and 38 males (Table 1). The age of respondents ranged between 18 to 87 in females and 16 to 83 in males, with a mean of 44 and 48 years, respectively.

Considering sampled households, 35 of the 57 interviewed (61.4%), reported that small ruminants were managed under a zero-grazing method, in which animals were stall-fed on grasses and food residues. In the remaining herds, 13 and 9 farmers reported to apply open-grazing and semi-zero grazing systems, respectively, in which the small ruminants were allowed to graze freely or go around grazing and get a supplement of food residues once back home. Of the 57 farms targeted, the biggest share (36 out of 57, representing 63.2%) was owning goats, whereas 9 (15.8%) farms had only sheep and 12 (21.1%) had both sheep and goats housed together at farm level. In interviewed farmers, majority (47 of the 57, 82.5%) reported that their animals were obtained from local livestock markets and others (10/57, making up 17.5%) through various donations. Interestingly, all farmers reported not to observe any quarantine period prior to introduction of new animals in their herds.

### Sampled Animals and Occurrence of Small Ruminant Diseases

On a period of 12 months, interviewed farmers reported occurrence of abortions in 19 of their farms, representing 33.3%, whereas 12 farmers (21%) indicated occurrence of abortion incidences in neighboring farms. According to the 19 farmers who experienced abortions, 18 cases occurred in goats whereas 1 case concerned sheep. Also, of the 12 abortion occurrences observed in neighboring farms, all were reportedly observed in goats. Moreover, 24 farms (42.1%) highlighted occurrence of death involving small ruminants at their own farms in the past 12 months, whereas 21 (36.8%) reported events of small ruminant deaths in neighboring farms (Table 1).

In total, 316 goats (201 females and 115 males) and 144 sheep (82 females and 62 males) were sampled. Based on age dentition, the goats were classified into three main categories of age: between 6 and 15 months (115 goats), 1.5–3 years (105 goats) and those being more than 3 years (96 goats). Similarly, age estimation in sheep showed that 54 were between 6 and 15 months, 53 were between 1.5 and 3 years, whereas 37 were over 3 years (Table 2).

**Table 2:** Characteristics of small ruminants sampled.

**Characteristics**		**Goats**	**Sheep**	**Total**
Sex	Male	115	62	177
	Female	201	82	283
	Total	316	144	460
Age	6–15 months	115	54	169
	1.5–3 years	105	53	158
	>3 years	96	37	133
	Total	316	144	460

### Seroprevalence of PPR

A total of 14.8% (68/460) samples from small ruminants, including 17.4% (25/144) from sheep and 13.6% (43/316) from goats were seropositive for antibodies to PPRV (Table 3 and Supplementary Figure 1). After adjusting to the test specificity and sensitivity, the overall animal-level estimated true prevalence was 15.1% (95% CI: 12.0–18.9), whereas species-level estimated true prevalence was 13.9% (95% CI: 10.3–18.3) and 17.8% (95% CI: 12.2–25.3) in goats and sheep, respectively. Of the 57 farms sampled, 35 had at least one animal seropositive (61.4, 95% CI: 48.4–72.9), giving an estimated farm-level true prevalence of 64.8% (95% CI: 50.9–77.0).

**Table 3:** Seroprevalence of PPR in Small Ruminants according to various disease risk factors.

	**Risk factors**	**Goats**				**Sheep**			
		**Total No. of samples**	**No. of positive samples**	**Sero-prevalence %**	**95% CI**	**Total No. of samples**	**No. of positive samples**	**Sero-prevalence %**	**95% CI**
District	Nyagatare	75	13	17.3	(9.6–27.8)	21	4	19.0	(5.4–41.9)
	Bugesera	59	8	13.6	(6.0–25.0)	44	6	13.6	(5.2–27.4)
	Kirehe	70	9	12.9	(6.1–23.0)	29	5	17.2	(5.9–35.8)
	Musanze	56	8	14.3	(6.4–26.2)	31	8	25.8	(11.9–44.6)
	Gicumbi	56	5	8.9	(3.0–19.6)	19	2	10.5	(1.3–33.1)
	Total	316	43	13.6	(10.0–17.9)	144	25	17.4	(11.6–24.6)
Sex	Male	115	18	15.7	(9.5–23.6)	62	11	17.7	(9.2–29.5)
	Female	201	25	12.4	(8.2–17.8)	82	14	17.1	(9.7–27.0)
	Total	316	43	13.6	(10.0–17.9)	144	25	17.4	(11.6–24.6)
Age	6–15 months	115	7	6.1	(2.5–12.2)	54	5	9.3	(3.1–20.3)
	1.5–3 years	105	17	16.2	(9.7–24.7)	53	12	22.6	(12.3–36.2)
	>3 years	96	19	19.8	(12.4–29.2)	37	8	21.6	(9.8–38.2)
	Total	316	43	13.6	(10.0–17.9)	144	25	17.4	(11.6–24.6)
Farming system	Zero grazing	155	18	11.6	(7.0–17.7)	67	14	20.9	(11.9–32.6)
	Semi-zero grazing	37	6	16.2	(6.2–32.0)	20	2	10.0	(1.2–31.7)
	Open grazing	124	19	15.3	(9.5–22.9)	57	9	15.8	(7.5–27.9)
	Total	316	43	13.6	(10.0–17.9)	144	25	17.4	(11.6–24.6)

## Discussion

Sheep and goats represented 26% of 58,580 metric tons of red meat that was produced in Rwanda in 2017 (37) and this figure is expected to raise to meet growing population. Small ruminants are mainly raised for income generation through sales, but also for meat, wool and manure used in crop fields.

Information generated from this study show that diseases affecting small ruminant and causing deaths and/or abortions are prevalent in the sampled regions. However, due to inadequate veterinary services penetration in rural Rwandan regions, characterized by widespread of less qualified veterinary paraprofessionals (VPP), inadequate veterinary supervision of the VPP (38, 39) and unavailability of supporting laboratory services, diseases that caused abortions were not clearly identified and/or communicated to farmers. PPR is known to cause abortions at all stages of the pregnancy (40). Among possible differential diagnosis, Rift Valley Fever (RVF), another disease that causes abortions in affected animals (41), must be taken into consideration as a possible factor associated to the episodes reported by farmers. In fact, Rwanda has had its first outbreak of RVF declared in 2018 (42) and cases were mainly identified in the eastern region of the country, part of our study area.

This study is the first one to report seroprevalence of PPR in Rwanda. Our laboratory analyses indicated an estimated overall true prevalence of 15.1% (95% CI: 12.0–18.9) and seropositivity of 13.9% (95% CI: 10.3–18.3) and 17.8% (95% CI: 12.2–25.3) in goats and sheep, respectively. These findings of PPRV-specific antibodies circulating in goats and sheep sampled in various areas, constitute evidence of exposure to the disease. Further studies are needed to provide more insights on the epidemiology of PPR in Rwanda. For instance, other investigations should provide more information on nation-wide prevalence in susceptible domestic and wildlife animals, risk factors associated to PPR prevalence and phylogenetic characterization of circulating viruses in an attempt to determine origin, spread and distribution of various virus lineages and risk factors in Rwanda.

Based on laboratory data from this cross-sectional study, we certainly can confirm the exposure of goats and sheep to PPRV in sampled regions. The comparisons of prevalence with regional findings, should be undertaken carefully. In addition, this study has estimated prevalence of PPR in goats and sheep using non-probability sampling methods. Therefore, it does not allow generalizing the findings at country and small ruminant population level.

PPR is a well-known disease in the region as shown by endemic as well as epidemic events having been reported in Uganda, Tanzania, Burundi and Democratic Republic of Congo (9, 19, 22, 24, 28, 29, 43). In addition to reports of major outbreak in the region, various retrospective serological analyses, showed positive antibodies to PPRV and confirmed the prevalence of the virus and its circulation before occurrence of all recent outbreaks in above countries (17, 19, 22). Therefore, Rwanda Veterinary Services should strengthen active surveillance mechanisms in order to fully investigate prevalence of the disease and adopt prevention measures before occurrence of large outbreaks in the country.

Future studies on PPR in Rwanda should depict a clearer picture of the epidemiology of the disease in Rwanda. For example, due to limitations inherent to this study, some unanswered questions were for example, the difference in the distribution of PPR across various regions and possible contribution of animal age, sex, and husbandry to the occurrence of PPR.

Our data suggests a correlation between the age of the sampled animals and seroprevalence status within age groups. In fact, small ruminants of more than 15 months were more affected than younger ones. This finding is in conformity with other studies on PPR (28, 44) and can be explained by the facts that older animals have had more exposure time to the virus, especially if this is endemic in the region. In addition, older animals tend to move far from their home in search of greener pastures and water bodies. Some study participants (10 of the 57 interviewed, Table 1) reported that their small ruminants were acquired through livestock donating initiatives. Therefore, we cannot rule out the possibility that the livestock were seropositive to PPRV, when gifted to farmers. The presence of antibodies in younger animals is however suggestive of recent virus circulation in Rwanda and this should be investigated further.

Phylogenetic investigations showed that the virus lineages II, III and IV are circulating in the region. Based on limited available sequences, the lineage III seems predominating in western Uganda, eastern DRC and Burundi with sequence similarities in some countries such as DRC and Burundi (19). In addition, the 2017 outbreak of PPR that occurred in various regions in Burundi, followed introduction of Boer goats from potentially infected regions of Uganda and the goats were transported through Tanzania suggesting transboundary movements as possible route of PPRV transmission (22). The regional virus circulation, added to report of PPRV at wildlife-livestock interface in Kabale, Kisoro and eastern DRC; regions close to Rwanda (19), puts an emphasize on the role of movements of livestock across transnational boundaries as well as the role of wildlife animals in the circulation and maintenance of PPRV in the region. Our findings suggest that PPR is prevalent in Rwandan regions close to neighboring countries and areas with recent PPR outbreak events. Rwanda is located in an area characterized by large livestock as well as wildlife populations. In addition, the region is known for important livestock trade between regional countries including Rwanda, and eastern part of DRC (45). The presence of livestock and wildlife and trade movements across countries, could have contributed to the introduction of PPR in north and west parts of Rwanda. Last but not least, Rwanda has experienced large movements of returning citizens from neighboring countries in 1994 and from Tanzania in 2007 as well as Burundian refugees in 2015 (46–48). These movements of people and their livestock could have contributed to the introduction of PPR in various regions of Rwanda. Molecular epidemiology studies and analysis of transboundary livestock movements could shed more insights on PPR epidemiology in Rwanda and the region.

As a preliminary report, this study has several limitations. First, although PPR has never been declared in Rwanda, samples were taken from places relatively close to the borders of the country with countries with known reports of PPR in past years. Due to possible more intense transboundary livestock movements in sampled areas than in other parts of the country, the prevalence found in this study may not necessarily reflect a country-large situation. To minimize this bias, the calculation of needed sample size, assumed a prevalence of 50% which is a condition that maximizes the sample size. Secondly, sampling methods were non-randomized and only a small number of farms was reached. Therefore, without assumption of a homogenous population, the data presented may not be representative of the entire population of small ruminant farmers. Therefore, the current findings do not allow to calculate the true prevalence, analyse the risk factors or to compare prevalence across the study areas. Third, cross-sectional surveys are not suitable for detection of rare, non-endemic diseases such as PPR with an unknown status in Rwanda.

Further studies are needed to collect representative evidence, informative for the eradication and control programs. In this regard, comprehensive studies using probabilistic sampling methods are recommended to investigate PPR at wildlife-livestock interface in Rwandan regions neighboring DRC, Uganda, Tanzania and Burundi. Such studies would help to follow up the occurrence of disease events in small ruminants, and would retrospectively collect evidence of possible endemicity of PPR. This is justified by the possibility of regional circulation of the virus along with livestock transboundary movements as hypothesized by previous studies (19, 22). Rwanda is home to natural parks and forests which may serve as PPRV hotspots at the interface of livestock and wildlife. Therefore, future studies in Rwanda and the region, must take into consideration the livestock and wildlife components, in order to fully understand the epidemiology of PPR.

## Data Availability Statement

The raw data supporting the conclusions of this article will be made available by the authors, without undue reservation.

## Ethics Statement

The studies involving human participants were reviewed and approved by Ethical approval for this study was obtained from the College of Agriculture, Animal Sciences and Veterinary Medicine, University of Rwanda (Ethical approval reference: 025/17/DRIPGS). The patients/participants provided their written informed consent to participate in this study.

## Author Contributions

AS contributed to conceptualization, laboratory analysis, data curation, investigation, methodology, and writing original draft. MU participated in conceptualization, investigation, methodology, and manuscript editing. JW contributed to conceptualization, funding acquisition, supervision, review of the manuscript, and editing conception and design of the study. All authors contributed to the article and approved the submitted version.

## Conflict of Interest

The authors declare that the research was conducted in the absence of any commercial or financial relationships that could be construed as a potential conflict of interest.

## Supplementary Material

Supplementary Figure 1 Percentage Inhibition (PI) distribution based on competitive ELISA results (ID screen^®^ PPR competition, IDvet Genetics, Grabels, France). The distributions are shown by sampled locations (District). Positive samples have below 50% PI and are shown in the graphs with a dark dashed black line.
