# Supplementary figures and images for: Serological Evidence of Exposure to Peste des Petits Ruminants in Small Ruminants in Rwanda
Source: Front Vet Sci. 2021 Mar 4;8:651978. doi: 10.3389/fvets.2021.651978 (PMC7970037; doi:10.3389/fvets.2021.651978)

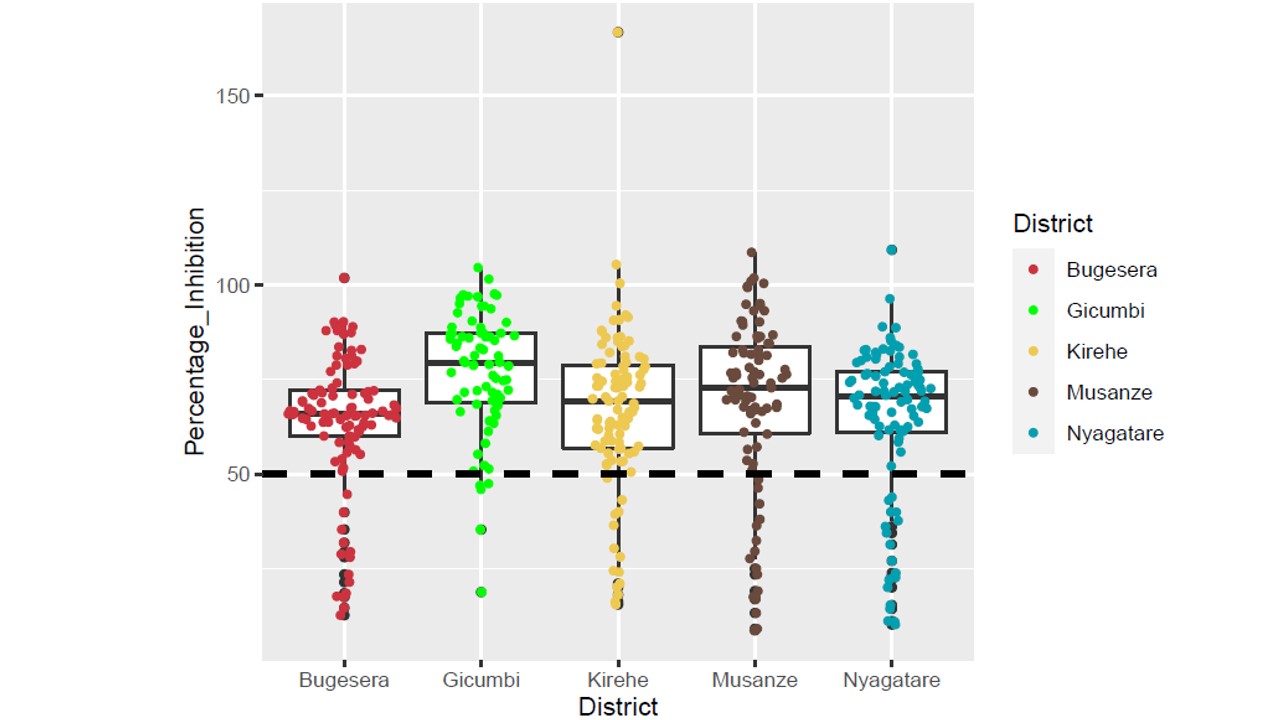

Supplement: Supplementary Figure 1 — Percentage Inhibition (PI) distribution based on competitive ELISA results (ID screen® PPR competition, IDvet Genetics, Grabels, France). The distributions are shown by sampled locations (District). Positive samples have below 50% PI and are shown in the graphs with a dark dashed black line. [file Image_1.jpg]
